# Patient attendance at a pediatric emergency referral hospital in an area with low COVID-19 incidence

**DOI:** 10.1371/journal.pone.0258478

**Published:** 2021-10-14

**Authors:** Koichi Shichijo, Shunsuke Takeuchi, Takahiro Tayama, Mikiko Takei, Keisuke Fujioka, Akemi Ono, Miki Shono, Kenichi Suga, Reiko Kondo, Takashi Kaimen, Nobuo Arima, Shuji Kondo

**Affiliations:** 1 Department of Pediatrics, Tokushima Prefectural Central Hospital, Tokushima, Japan; 2 Department of Pediatrics, Tokushima University Hospital, Tokushima, Japan; 3 Department of Psychiatry, Tokushima Prefectural Central Hospital, Tokushima, Japan; 4 Patient Support Center, Tokushima Prefectural Central Hospital, Tokushima, Japan; University of South Carolina, UNITED STATES

## Abstract

The purpose of this study was to clarify the effects of individual infection control measures and physical distancing on pediatric medical care in a local prefecture in Japan, where the incidence of coronavirus disease (COVID-19) in pediatric patients was extremely low. We extracted data from hospital records on the number of outpatients, inpatients, infectious disease consultations, and consultations for representative pediatric diseases. We compared attendance in 2017–2019, before the COVID-19 pandemic, with 2020, when COVID-19 spread to Japan. There were no COVID-19 patients in the pediatric department during the study period. The total number outpatient visits decreased by 24.4%, and the number of hospital admissions, excluding neonatal care unit admissions, decreased by approximately 35%. There was a marked reduction in the number of hospitalizations for infectious diseases such as influenza (−74.8%) and respiratory syncytial virus infection (−93.5%), and the number of hospitalizations for bronchitis/pneumonia, Kawasaki disease, and bronchial asthma decreased. In contrast, the number of clinical psychological interventions and cases reported to the child guidance center increased. In the context of pandemic infectious diseases, it is important to control the spread of problematic infectious diseases by individual infection control measures and physical distancing. However, it is necessary to maintain social life as much as possible for the mental health and physical development of children.

## Introduction

Coronavirus disease (COVID-19), which is caused by severe acute respiratory syndrome coronavirus 2 (SARS-CoV-2), was confirmed in Wuhan in the People’s Republic of China in December, 2019 and subsequently spread globally [1]. The first case of domestic infection occurred in Japan on January 15, 2020. In response to an increasing number of COVID-19 cases, the Japanese government issued an emergency declaration on April 7, 2020, leading to a lockdown limited to large cities [2]. The lockdown was extended nationwide on April 16 and continued until May 25 [3]. Although many countries, including several European countries, carried out a strict lockdown during the COVID-19 pandemic, the lockdown in Japan was mild compared with some other countries. Each prefectural governor asked the residents to refrain from going out unnecessarily, and recommended that people and companies restrict the use of stores and facilities and work remotely. These measures were voluntary [4]. All schools were closed except for infant daycare centers and nursery schools from March 2 to the end of May. At the same time, the public was encouraged to take infection control measures such as using face masks and hand washing, and to avoid the “three Cs” (Closed spaces, Crowded places, and Cross-contact settings) by physical distancing [5]. The number of outpatient visits, not only for adults but also for children, is reported to have decreased in emergency hospitals after the spread of COVID-19 [6–8]. These reports compared the number of emergency patients during the lockdown period with the same period in the previous year. It has not been determined whether the decreased number of patients was due to the strict lockdown or to the suppression of infectious diseases as a result of infection control measures and physical distancing.

This study aimed to clarify the effects of individual infection control measures and physical distancing on pediatric medical care, including the number of outpatients, inpatients, infectious disease diagnoses, and other representative diseases, at a pediatric emergency referral hospital in a local prefecture in Japan, where strict lockdown was not implemented and the incidence of pediatric COVID-19 was extremely low.

## Materials and methods

This study was an observational descriptive study of yearly and monthly pediatric patients at the Tokushima Prefectural Central Hospital before and after the onset of the COVID-19 pandemic. This study was conducted with the approval of the Tokushima Prefectural Central Hospital Ethics Committee (approval number 20–26). We accessed the data from January 2021 to February 2021. All data were fully anonymized before we accessed them. The requirement for informed consent was waived because the study was a retrospective study.

### Participants

All patients who received medical care in the hospital pediatrics department from January 1, 2017, to December 31, 2020, were included in the study. We extracted data for the study period from medical records. We analyzed the total number of pediatric outpatients, the number of pediatric emergency consultations, the number of general pediatric inpatients, excluding patients in the neonatal intensive care unit (NICU) and growing care unit (GCU).

Tokushima Prefecture is located on the eastern side of Shikoku Island in Japan. As of July 1, 2020, the total population of Tokushima Prefecture was 722,653, and the population of children less than 15 years old was 80,202 (41,242 male and 38,960 female) [9]. The hospital is located in Tokushima City, the central city of the prefecture, and plays a role as a pediatric emergency referral hospital. In principle, the pediatrics department provides medical care for patients under the age of 15 years. The hospital accepts pediatric referral patients and emergency transport patients from throughout Tokushima Prefecture 24 hours a day. The hospital accepts mostly secondary and tertiary emergency patients after hours from 17:00 to 8:30 the following day on weekdays, and the full day on weekends and holidays. After hours primary emergency patients are treated at some pediatric private clinics and holiday-night clinics established by Tokushima City. However, the pediatrics department also accepts primary emergency patients from 22:30 to 8:30 the following day because of the closure of all the primary care clinics. The department does not provide surgical care for trauma because these patients are treated by the general emergency doctors. The hospital cares for newborns born after 30 weeks of gestation, accepts transfers of pregnant women and newborns from neighboring obstetric medical institutions, and provides medical care in the NICU and the GCU. In addition, the hospital established a medical care system to accept and treat both adults and children with COVID-19 as the main public medical institution in the prefecture.

### Data collection and analysis

Data were extracted from the medical records of pediatric patients. We performed a descriptive analysis using the Microsoft Excel (Microsoft Corporation, Redmond, WA) software, version 2019. The following five items were analyzed:

#1 Changes in the number of outpatients;
#2 Changes in the number of inpatients;
#3 Changes in the number of infectious diseases;
#4 Changes in the number of inpatients with representative pediatric diseases;
#5 Changes in the numbers of clinical psychological interventions and cases reported to the child guidance center.

We calculated the annual number of the cases for each year from 2017‌–2020. We compared each item in 2017−2019, before COVID-19 pandemic, and in 2020, when COVID-19 was reported in Japan. In addition, we compared the average number of patients in 2017‌–2019 with that in 2020.

For #1, we counted the total number of pediatric outpatients, pediatric emergency patients, and pediatric referrals. For #2, we counted the number of general pediatric, NICU, and GCU inpatients. For items #1 and #2, monthly data were examined in addition to the total number for one year. For #3, the hospital is an influenza sentinel surveillance site and a National Epidemiological Surveillance of Infectious Diseases site, which is an epidemiological survey system for infectious diseases in Japan. Surveillance includes influenza, respiratory syncytial virus (RSV) infection, pharyngoconjunctival fever, group A streptococcal pharyngitis, infectious gastroenteritis, chickenpox, hand-foot-and-mouth disease, erythema infectiosum, exanthem subitum (roseola infantum), herpangina, and mumps. Influenza included types A and B. Infectious gastroenteritis was limited to viral infections. We used these surveillance data as annual data. Monthly data were extracted for influenza, RSV infection, and infectious gastroenteritis, which are all common infections. We performed antigen and nucleic acid amplification tests for SARS-CoV-2 when there was a possibility that the patient had SARS-CoV-2 infection. For #4, we investigated bronchitis/pneumonia, Kawasaki disease, status epilepticus, bronchial asthma, and urinary tract infections, which are representative diseases that are common causes of hospitalization in children. For #5, we first counted the total number of patients who were referred to the clinical psychologists for psychological interventions. Children with physical symptoms such as chest pain, abdominal pain, and visual impairment, but no abnormalities detected on examination were judged to be psychophysiological disorders. We also examined suicide attempts and child deaths. We next counted the number of cases of suspected child abuse based on information about abusive trauma, including subdural hematoma, bleeding in the fundus of the eye, head fracture, and neglect. Such cases are required to be reported to the child guidance center through medical social workers. Of these, the number of abusive head trauma (AHT) in infants and children was counted as an indicator of severe forms of child abuse.

## Results

### Changes in the number of outpatients

**Fig 1:**
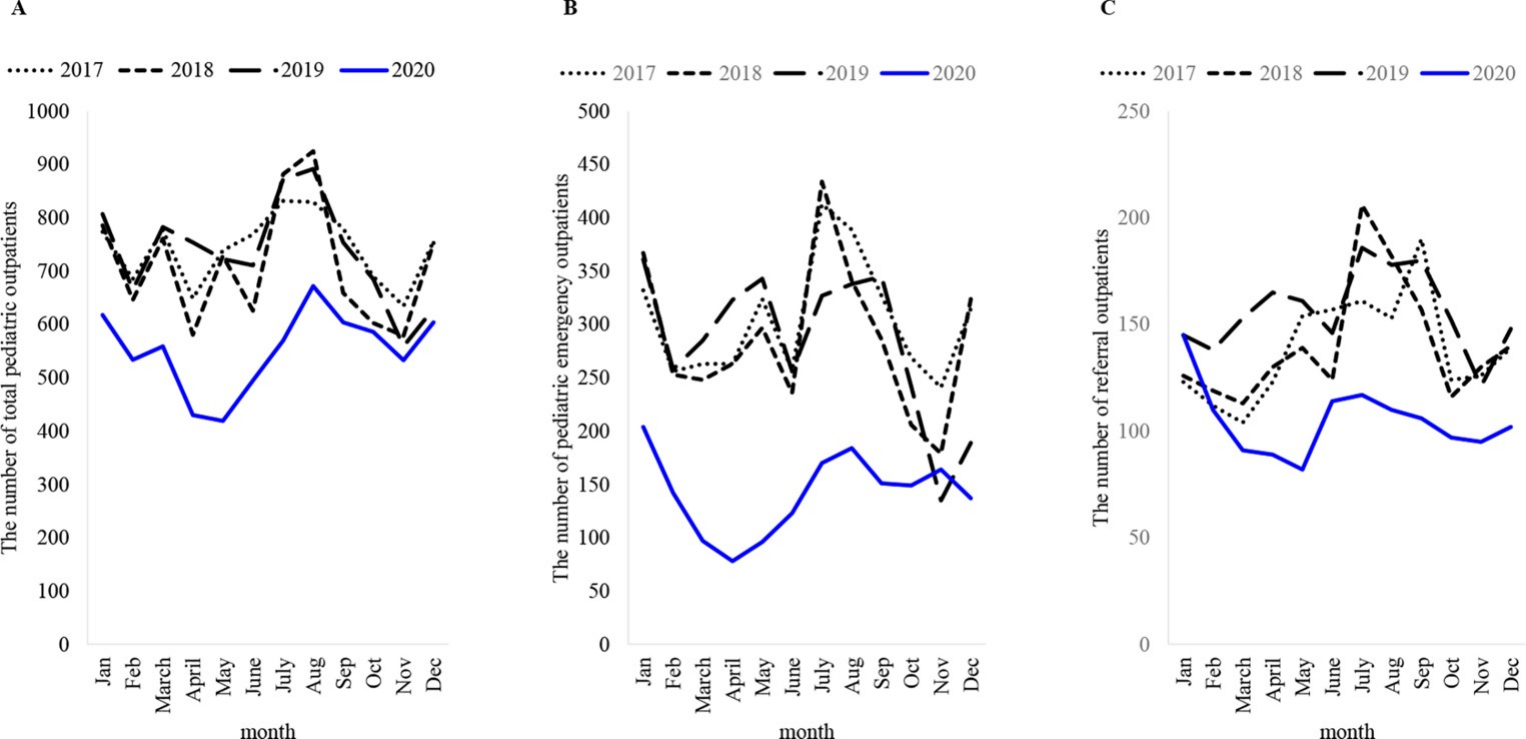
Changes in the number of outpatients by month and year. A: Total pediatric outpatients; B: Pediatric emergency outpatients; C: Referral outpatients. (S1–S3 Tables).

As shown in Table 1, the total number of pediatric outpatients exceeded 8000 per year in 2017–2019, but it decreased to 6624 (−24.4%) in 2020. The number of pediatric emergency patients exceeded 3000 per year in 2017–2019 but decreased markedly to 1695 (−51.5%) in 2020. The number of pediatric referrals exceeded 1600 per year in 2017–2019 but decreased to 1258 (−27.7%) in 2020. The tendency for a greater number of male patients than female patients remained consistent throughout the study period. There was also no change observed in the age distribution in 2020 compared with 2017–2019, although the decline of patients over 10 years of age was less marked. Monthly data on the number of total pediatric outpatients showed a constant decline throughout the year 2020 (Fig 1A). In particular, the number of emergency patients strongly declined during the lockdown period and was sustained thereafter (Fig 1B). The number of patient referrals declined during the lockdown period and has continued to decrease after July; however, the decline was less marked than the decline in emergency patients (Fig 1C). We had no remote or telephone consultation practice.

**Table 1:** Yearly changes in the number of outpatients.

	2017	2018	2019	2020	% Change
**Total pediatric outpatients**	8916	8530	8836	6624	−24.4
Age (years), n (%)					
0–4	5508 (62)	5202 (61)	5223 (59)	3827 (58)	−27.9
5–9	1803 (20)	1709 (20)	1902 (22)	1325 (20)	−26.6
10–15	1375 (15)	1405 (16)	1517 (17)	1306 (20)	−8.8
≥16	230 (3)	214 (3)	194 (2)	166 (3)	−22.1
Female sex, n (%)	3805 (43)	3619 (42)	3849 (44)	2961 (45)	
**Pediatric emergency outpatients**	3649	3434	3402	1695	−51.5
Age (years), n (%)					
0–4	2569 (70)	2396 (70)	2305 (68)	1165 (69)	−51.9
5–9	698 (19)	612 (18)	703 (21)	299 (18)	−55.4
10–15	374 (10)	420 (12)	388 (11)	226 (13)	−42.6
≥16	8 (0)	6 (0)	6 (0)	5 (0)	−25.4
Female sex, n (%)	1559 (43)	1522 (44)	1433 (42)	741 (44)	
**Referral outpatients**	1666	1682	1873	1258	−27.7
Age (years), n (%)					
0–4	1136(68)	1192(71)	1261(67)	803(64)	−32.9
5–9	308(18)	280(17)	362(19)	257(20)	−18.9
10–15	214(13)	205(12)	242(13)	191(15)	−13.2
≥16	8(0)	5(0)	8(0)	7(1)	0
Female sex, n (%)	708(42)	726(43)	804(43)	553(44)	

% Change means the change from the average number of patients in 2017‌–2019 to that in 2020.

### Changes in the number of inpatients

**Fig 2:**
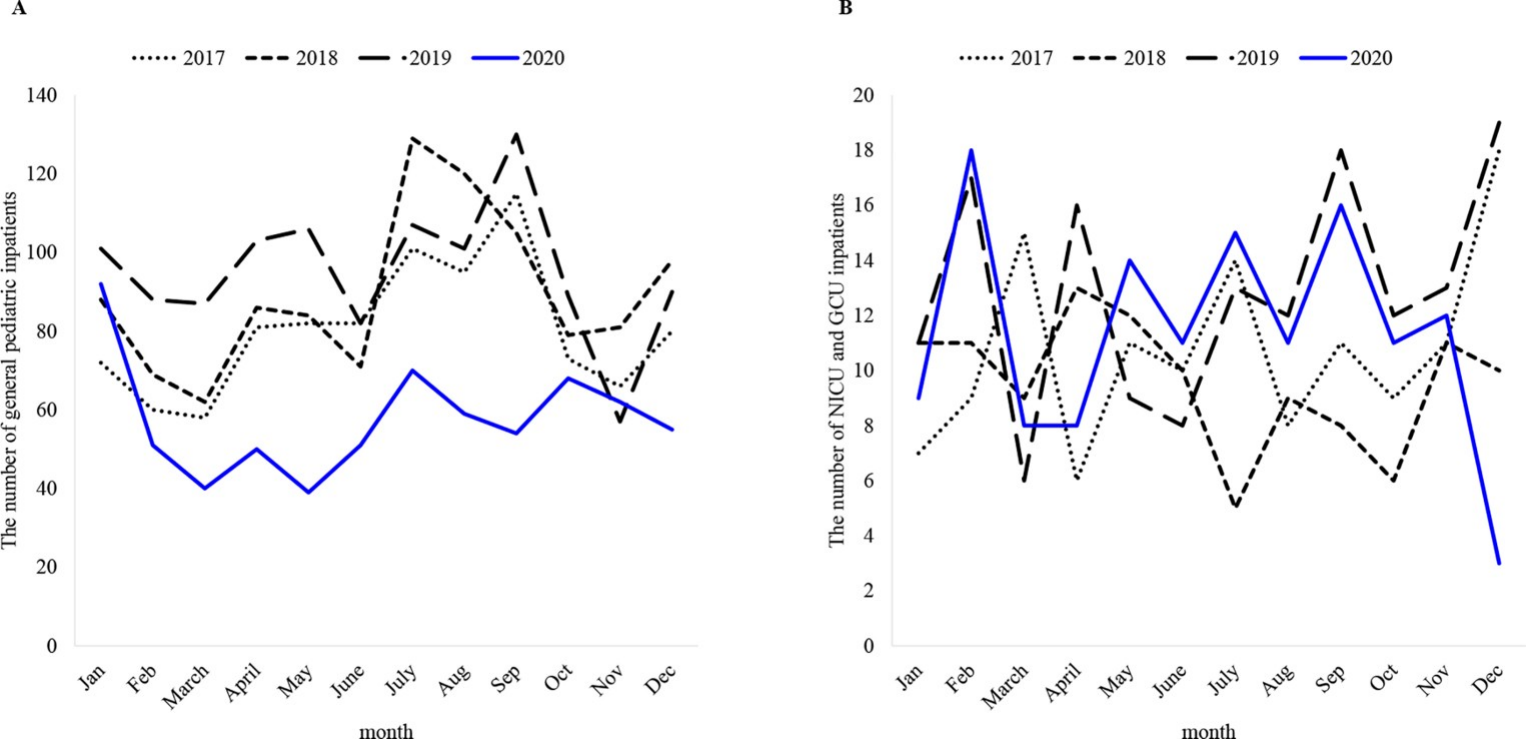
Changes in the number of inpatients by month and year. A: General pediatric inpatients; B: NICU and GCU inpatients. NICU: neonatal intensive care unit; GCU: growing care unit. (S4 and S5 Tables).

As shown in Table 2, the number of general pediatric inpatients was around 1000 in 2017–2019 but decreased to 691 (−34.7%) in 2020. Monthly data on general pediatric inpatients showed a decline in all months except November (Fig 2A). The number of NICU and GCU inpatients in 2020 was slightly lower than that in 2019, but it was higher than that in 2017 and 2018. The decrease by three cases in December 2020 was due to the limited number of beds available and long-term room occupancy by three very low birthweight babies who required care in the NICU for an extended period (Fig 2B). The tendency for a slight preponderance of males to be treated in the pediatric ward, the NICU, and the GCU did not change during the study period.

**Table 2:** Yearly changes in the number of inpatients.

	2017	2018	2019	2020	% Change
**General pediatric inpatients**	965	1072	1141	691	−34.7
Age (years), n (%)					
0–4	741 (77)	843 (79)	837 (73)	485 (70)	−39.9
5–9	147 (15)	134 (13)	193 (17)	128 (19)	−19.0
10–15	71 (7)	94 (9)	107 (9)	76 (11)	−16.5
≥16	6 (1)	1 (0)	4 (0)	2 (0)	−45.9
Female sex, n (%)	391 (41)	435 (41)	488 (43)	292 (42)	
**NICU/GCU inpatients**	129	115	154	136	+2.5
Female sex, n (%)	57 (44)	48 (42)	53 (34)	63 (46)	

% Change means the change from the average number of patients in 2017‌–2019 to that in 2020.

### Changes in the number of infectious diseases

**Fig 3:**
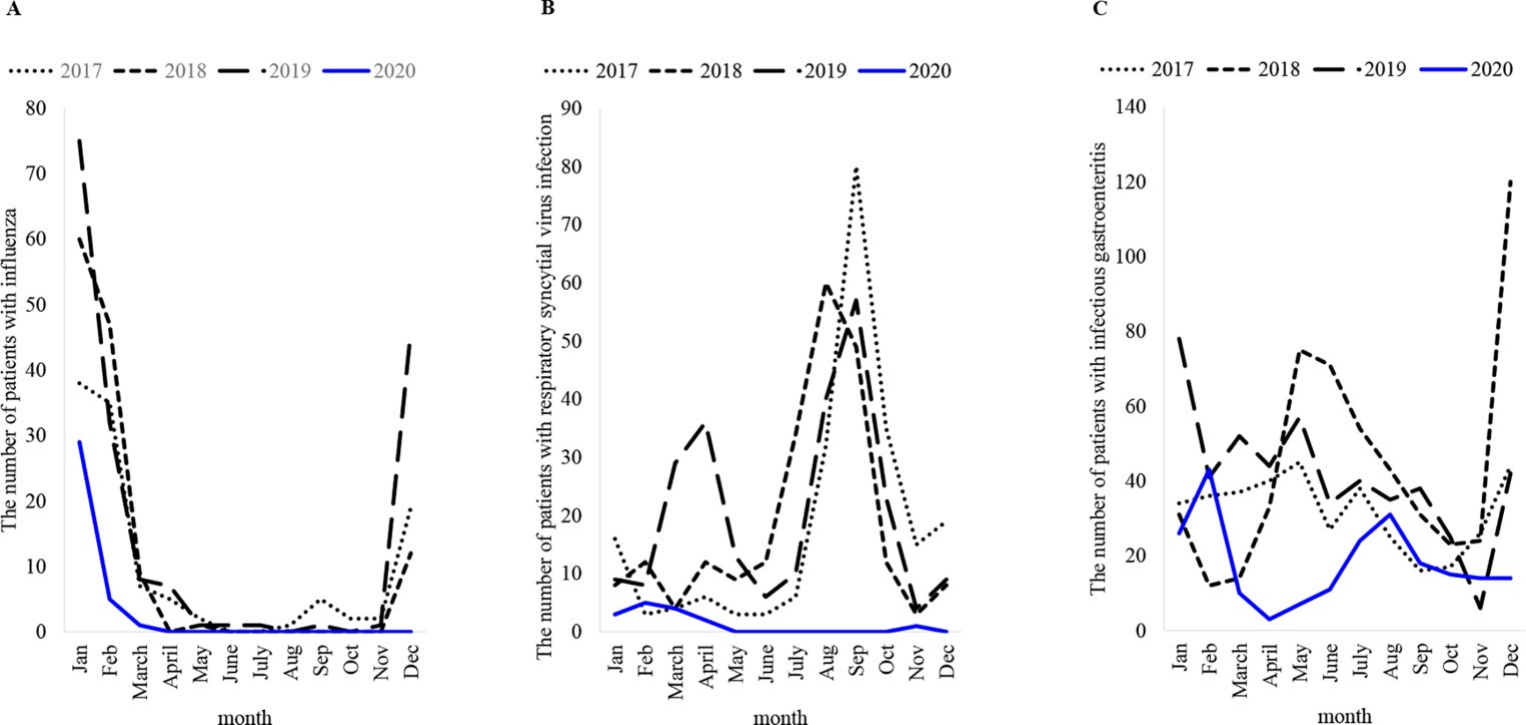
Changes in the number of cases of influenza, respiratory syncytial virus infection, and infectious gastroenteritis by month and year. A: influenza. B: respiratory syncytial virus infection. C: infectious gastroenteritis. (S6–S8 Tables).

The annual number of several indicator infectious diseases is shown in Table 3. There were >100 cases of influenza each year in 2017–2019, but has decreased to 35 cases (−74.8%) in 2020. In 2020, there were 29 cases of influenza in January, five in February and one in March. There were no outpatient or inpatient cases of influenza from April to the end of December 2020 (Fig 3A). The number of cases of RSV infection was >200 per year in 2017–2019 but showed a marked decline to 15 (−93.5%) in 2020. In 2020, there was only one patient with RSV after July when the number of patients usually begins to increase (Fig 3B). There were no patients with hand-foot-and-mouth disease, which is commonly seen from June to August. The number of cases of herpangina also decreased from 26 cases in 2019 to two in 2020 (−88.9%). There were 385–531 cases of infectious gastroenteritis per year in 2017–2019, but the number of cases decreased to 216 (−53.9%) in 2020. Specifically, the incidence of infectious gastroenteritis was lower from April to June and in December compared to the monthly data from to 2017–2019 (Fig 3C). Group A streptococcal pharyngitis occurred in 13 cases (−7.1%), which was similar to the previous year. The number of cases of exanthem subitum increased by 82.3% from 2017–2019. In addition, there were no cases of pharyngoconjunctival fever, chickenpox, erythema infectiosum, or mumps in 2020. There were no cases of pediatric SARS-CoV-2 infection.

**Table 3:** Yearly changes in the number of infectious diseases.

	2017	2018	2019	2020	% Change
Influenza	116	129	173	35	−74.8
RSV infection	222	223	244	15	−93.5
Pharyngoconjunctival fever	3	0	0	0	N/A
GAS pharyngitis	17	14	11	13	−7.1
Infectious gastroenteritis	385	531	492	216	−53.9
Chickenpox	0	1	1	0	N/A
Hand-foot-and-mouth disease	32	9	26	0	N/A
Erythema infectiosum	0	0	3	0	N/A
Exanthem subitum	23	16	22	37	+82.3
Herpangina	12	16	26	2	−88.9
Mumps	20	2	1	0	N/A

% Change means the change from the average number of patients in 2017‌–2019 to that in 2020.

GAS, group A streptococcus; N/A, not applicable; RSV, respiratory syncytial virus.

### Changes in the number of inpatients with representative pediatric disease

There were >100 cases of bronchitis/pneumonia per year in 2017–2019, but the number of cases decreased to 44 (−74.2%) in 2020. There were 65–72 cases of Kawasaki disease per year in in 2017–2019, but this decreased to 47 cases (−31.2%) in 2020. Status epilepticus was 15–26 cases in the past 3 years, but increased to 32 cases (+54.6%) in 2020. Bronchial asthma decreased from to 39–46 cases to 19 cases (−55.1%). There was no marked change in the number of cases of urinary tract infection (Table 4).

**Table 4:** Yearly changes in the number of inpatients with representative pediatric disease.

	2017	2018	2019	2020	% Change
Bronchitis/Pneumonia	122	246	144	44	−74.2
Kawasaki disease	68	65	72	47	−31.2
Status epilepticus	26	15	21	32	+54.6
Bronchial asthma	42	39	46	19	−55.1
Urinary tract infection	26	35	33	26	−16.9

% Change means the change from the average number of patients in 2017‌–2019 to that in 2020.

### Changes in the numbers of clinical psychological interventions and cases reported to the child guidance center

Clinical psychological intervention was started in 2018. Patients were more likely to be female and tended to be more than 10 years old (Table 5). The number of psychological interventions was 211 (+257.6%), and the number of new patients increased to 19 (+72.7%) in 2020. There were two suicide attempts in 2018, one in 2019, and one in 2020 but no completed suicide throughout the study period. There were 2, 2, 1, and 2 cases of child death in 2017, 2018, 2019, and 2020, respectively. The number of cases reported to the child guidance center increased to 11 (+64.2%) in 2020. Eight out of 11 cases were children under 4 years old, and half of them were AHT in 2020.

**Table 5:** Yearly changes in the numbers of clinical psychological interventions and cases reported to the child guidance center.

	2017	2018	2019	2020	% Change
**Clinical psychological interventions**	-	46	72	211	+257.6
New patient	-	11	11	19	+72.7
Age (years), n (%)	-				
0–4	-	0 (0)	1 (9)	1(5)	+100.0
5–9	-	2 (18)	1 (9)	3 (16)	+100.0
10–15	-	8 (73)	9 (82)	14 (74)	+64.7
≥16	-	1 (9)	0 (0)	1 (5)	+100.0
Female sex, n (%)	-	6 (55)	9 (82)	15 (79)	
**Cases reported to the child guidance center**	8	4	8	11	+64.2
Age (years), n (%)					
0–4	5 (63)	3 (75)	6 (75)	8 (73)	+70.2
5–9	2 (25)	0 (0)	0 (0)	0 (0)	N/A
10–15	1 (13)	1 (25)	2 (25)	2 (18)	+53.8
≥16	0 (0)	0 (0)	0 (0)	1 (9)	N/A
Female sex, n (%)	2 (25)	2 (50)	4 (50)	6 (55)	
AHT	1	0	1	4	+497.0

% Change means the change from the average number of patients in 2017‌–2019 to that in 2020.

AHT, abusive head trauma; N/A, not applicable.

## Discussion

There were no cases of SARS-CoV-2 infection seen in the pediatrics department during the study period. According to the Tokushima Prefecture authorities, there were only two cases of COVID-19 in children under the age of 15 years reported in Tokushima Prefecture in 2020 [10], and 199 cases in the prefecture overall (0.03% of the total population). Compared with the incidence of COVID-19 in many other countries and the urban regions of Japan, the COVID-19 epidemic within the prefecture was light in 2020 [11, 12].

The present study demonstrated that both the number of outpatients and inpatients decreased remarkably in a setting where COVID-19 was controlled. To clarify the cause, we investigated changes in infectious diseases and representative diseases of pediatric inpatients. The incidence of infectious diseases, such as influenza, RSV infection, hand-foot-and-mouth disease, and herpangina, were very low after the start of the COVID-19 pandemic. These findings are consistent with previous reports showing reductions of respiratory infections in the first wave of the COVID-19 pandemic [13, 14]. The decrease in infectious disease is likely to be due to individual infection control measures such as the use of face masks, hand washing, and physical distancing. The reduction of bronchitis/pneumonia and bronchial asthma may be attributable to the decrease in infectious diseases, suggesting that these conditions are triggered and exacerbated by infectious diseases. The number of patients with Kawasaki disease also decreased. The association between the decline of Kawasaki disease patients and the decreased incidence of infectious diseases suggests that vasculitis, as occurs in Kawasaki disease, might be triggered by infectious diseases. Multisystem inflammatory syndrome in children (MIS-C) shares some symptoms with Kawasaki disease and is triggered by SARS-CoV-2 infection. In contrast to Kawasaki disease, MIS–C has been reported more frequently among children with COVID-19 in Europe and the United States than in Asian countries, where the incidence of MIS-C is low [15]. It is necessary to elucidate the pathogenic mechanism of Kawasaki disease and MIS-C, including genetic susceptibility [15, 16]. The degree of reduction in urinary tract infection was not obvious. Considering the large decrease in respiratory infections, our data was coincident with a relatively increased prevalence of urinary tract infections among pediatric fever patients in Taiwan [17]. The number of inpatients due to status epilepticus, which is often induced by high fever based on infectious diseases, increased 1.5-fold. The increased incidence of status epilepticus may be partly related to an increase in children with exanthem subitum at the hospital. The total number of exanthem subitum cases reported in Tokushima Prefecture was similar to the previous year [18]. Horizontal transmission from parents is considered one of the transmission routes for exanthem subitum [19]. In addition, infants under 2 years of age did not need to wear face masks and could maintain their social group life at infant daycare centers and nursery schools without physical distancing. The maintenance of opportunities for physical contact may account for the lack of decrease in the number of patients with status epilepticus and exanthem subitum. The number of inpatients in the neonatal care unit did not change, indicating that the neonatal care unit was not directly affected by the decline of infectious diseases. However, the number of pregnancies in Japan has decreased since the start of the COVID-19 pandemic, and the number of births is expected to decrease in the near future [20].

While infectious diseases have decreased, the number of clinical psychological interventions has tripled. Since psychosomatic disorders are included in pediatric emergency patients, clinical psychological counseling was introduced at the hospital in 2018, and its demand has been increasing. However, the number of the latest psychological interventions notably increased compared with the increased number in 2019. In Japan, school closures were introduced nationwide on March 2, 2020, and continued until the end of May [21]. It has been reported that children who have experienced social isolation by home confinement under school closure to prevent the spread of SARS-CoV-2 may be more prone to depression [22]. The number of cases reported to the child guidance center increased slightly, but this increase is difficult to interpret because of the low number of cases in the present study. In 2020, there were more cases of AHT, a severe type of child abuse, with subdural hematoma and fundus hemorrhage. Centers for Disease Control and Prevention in the United States reports that the COVID-19 pandemic has increased the proportion of patients with child abuse and neglect requiring hospitalization in the emergency department [23]. We speculate that the suppression of social life has had a significant negative impact on the psychological status of children in addition to their parents. Maintaining the appropriate social life of children and their families to the extent that it is possible, while taking infection control measures and physical distancing, is important for mental health and physical development [4].

The present study has some limitations. First, the study was conducted at a single institution. Thus, children with illness might refrain from visiting the hospital or consult other hospitals due to fear of acquiring SARS-CoV-2 infection. However, it is supposed that a similar phenomenon occurred at other medical institutions because there was no epidemic of influenza or RSV infection in Tokushima Prefecture [18]. Second, we were unable to investigate how lifestyle diseases and chronic diseases changed because the pediatric emergency referral hospital mainly treats children with acute symptoms. The number of children with lifestyle diseases, such as obesity and hypertension, might increase due to the lack of exercise and overeating under social life restraint. The incidence of chronic diseases, such as kidney disease and malignancy, might also change. Because the measures for infection control and social life control are very different in each country, the effects of the COVID-19 pandemic on the incidence and types of disease affected is likely to differ according to the country. Further epidemiological studies could help clarify the relationship between specific infection control measures and the incidence and spectrum of disease.

## Conclusion

With individual infection control measures and physical distancing, the number of outpatients, inpatients, and the incidence of infectious diseases and indicator diseases in children has decreased significantly in an area where the incidence of COVID-19 is low. In contrast, both numbers of clinical psychological interventions and cases reported to the child guidance center increased. It is necessary to maintain the quality of social life for children as much as possible for mental health and physical development while observing infection control measures and physical distancing.

## Supporting information

S1 Table Changes in the number of outpatients by month and year: Total pediatric outpatients. (PDF)

S2 Table Changes in the number of outpatients by month and year: Pediatric emergency outpatients. (PDF)

S3 Table Changes in the number of outpatients by month and year: Referral outpatients. (PDF)

S4 Table Changes in the number of inpatients by month and year: General pediatric inpatients. (PDF)

S5 Table Changes in the number of inpatients by month and year: NICU and GCU inpatients. (PDF)

S6 Table Changes in the number of cases of influenza by month and year. (PDF)

S7 Table Changes in the number of cases of respiratory syncytial virus infection by month and year. (PDF)

S8 Table Changes in the number of cases of infectious gastroenteritis by month and year. (PDF)
