# Supplementary material for: Patient attendance at a pediatric emergency referral hospital in an area with low COVID-19 incidence
Source: PLoS One. 2021 Oct 14;16(10):e0258478. doi: 10.1371/journal.pone.0258478 (PMC8516272; doi:10.1371/journal.pone.0258478)
Supplement: S1 Table — (PDF) [file pone.0258478.s001.pdf]

S1 Table. Changes in the number of outpatients by month and year: Total pediatric outpatients.

	2017	2018	2019	2020
Jan	774	786	807	618
Feb	685	646	665	534
March	777	761	783	559
April	649	581	754	430
May	739	729	723	419
June	769	626	711	495
July	832	882	873	570
Aug	830	925	892	672
Sep	780	659	754	604
Oct	690	603	685	586
Nov	635	579	559	533
Dec	756	753	630	604
