# Supplementary material for: Patient attendance at a pediatric emergency referral hospital in an area with low COVID-19 incidence
Source: PLoS One. 2021 Oct 14;16(10):e0258478. doi: 10.1371/journal.pone.0258478 (PMC8516272; doi:10.1371/journal.pone.0258478)
Supplement: S2 Table — (PDF) [file pone.0258478.s002.pdf]

S2 Table. Changes in the number of outpatients by month and year: Pediatric emergency outpatients

	2017	2018	2019	2020
Jan	332	367	361	204
Feb	256	253	258	142
March	263	248	285	97
April	263	263	323	78
May	324	297	343	96
June	257	236	256	123
July	413	434	327	170
Aug	389	341	338	184
Sep	327	286	345	151
Oct	269	206	242	149
Nov	241	179	135	164
Dec	315	324	189	137
