# Supplementary material for: Patient attendance at a pediatric emergency referral hospital in an area with low COVID-19 incidence
Source: PLoS One. 2021 Oct 14;16(10):e0258478. doi: 10.1371/journal.pone.0258478 (PMC8516272; doi:10.1371/journal.pone.0258478)
Supplement: S3 Table — (PDF) [file pone.0258478.s003.pdf]

S3 Table. Changes in the number of outpatients by month and year: Referral outpatients.

	2017	2018	2019	2020
Jan	123	126	145	145
Feb	112	119	138	110
March	104	113	153	91
April	123	130	165	89
May	154	139	161	82
June	157	124	146	114
July	161	206	186	117
Aug	153	182	178	110
Sep	190	157	180	106
Oct	124	116	152	97
Nov	126	130	121	95
Dec	139	140	148	102
