# Supplementary material for: Patient attendance at a pediatric emergency referral hospital in an area with low COVID-19 incidence
Source: PLoS One. 2021 Oct 14;16(10):e0258478. doi: 10.1371/journal.pone.0258478 (PMC8516272; doi:10.1371/journal.pone.0258478)
Supplement: S4 Table — (PDF) [file pone.0258478.s004.pdf]

S4 Table. Changes in the number of inpatients by month and year: General pediatric inpatients.

	2017	2018	2019	2020
Jan	72	88	101	92
Feb	60	69	88	51
March	58	62	87	40
April	81	86	103	50
May	82	84	106	39
June	82	71	82	51
July	101	129	107	70
Aug	95	120	101	59
Sep	115	105	130	54
Oct	73	79	89	68
Nov	66	81	57	62
Dec	80	98	90	55
