# Supplementary material for: Patient attendance at a pediatric emergency referral hospital in an area with low COVID-19 incidence
Source: PLoS One. 2021 Oct 14;16(10):e0258478. doi: 10.1371/journal.pone.0258478 (PMC8516272; doi:10.1371/journal.pone.0258478)
Supplement: S5 Table — (PDF) [file pone.0258478.s005.pdf]

S5 Table. Changes in the number of inpatients by month and year: NICU and GCU inpatients.

	2017	2018	2019	2020
Jan	7	11	11	9
Feb	9	11	17	18
March	15	9	6	8
April	6	13	16	8
May	11	12	9	14
June	10	10	8	11
July	14	5	13	15
Aug	8	9	12	11
Sep	11	8	18	16
Oct	9	6	12	11
Nov	11	11	13	12
Dec	18	10	19	3
