# Supplementary material for: Patient attendance at a pediatric emergency referral hospital in an area with low COVID-19 incidence
Source: PLoS One. 2021 Oct 14;16(10):e0258478. doi: 10.1371/journal.pone.0258478 (PMC8516272; doi:10.1371/journal.pone.0258478)
Supplement: S6 Table — (PDF) [file pone.0258478.s006.pdf]

S6 Table. Changes in the number of cases of influenza by month and year.

	2017	2018	2019	2020
Jan	38	60	75	29
Feb	35	47	32	5
March	7	9	8	1
April	5	0	7	0
May	2	1	1	0
June	0	0	1	0
July	0	0	1	0
Aug	1	0	0	0
Sep	5	0	1	0
Oct	2	0	0	0
Nov	2	0	1	0
Dec	19	12	46	0
