# Supplementary material for: Patient attendance at a pediatric emergency referral hospital in an area with low COVID-19 incidence
Source: PLoS One. 2021 Oct 14;16(10):e0258478. doi: 10.1371/journal.pone.0258478 (PMC8516272; doi:10.1371/journal.pone.0258478)
Supplement: S7 Table — (PDF) [file pone.0258478.s007.pdf]

S7 Table. Changes in the number of cases of respiratory syncytial virus infection by month and year.

	2017	2018	2019	2020
Jan	16	8	9	3
Feb	3	12	8	5
March	4	4	29	4
April	6	12	36	2
May	3	9	13	0
June	3	12	6	0
July	6	34	10	0
Aug	32	60	40	0
Sep	80	49	57	0
Oct	35	12	23	0
Nov	15	3	4	1
Dec	19	8	9	0
