# Supplementary material for: Patient attendance at a pediatric emergency referral hospital in an area with low COVID-19 incidence
Source: PLoS One. 2021 Oct 14;16(10):e0258478. doi: 10.1371/journal.pone.0258478 (PMC8516272; doi:10.1371/journal.pone.0258478)
Supplement: S8 Table — (PDF) [file pone.0258478.s008.pdf]

S8 Table. Changes in the number of cases of infectious gastroenteritis by month and year.

	2017	2018	2019	2020
Jan	34	31	78	26
Feb	36	12	41	43
March	37	14	52	10
April	40	33	44	3
May	45	75	57	7
June	27	71	34	11
July	38	54	40	24
Aug	25	43	35	31
Sep	16	31	38	18
Oct	17	23	25	15
Nov	26	24	6	14
Dec	44	120	42	14
